# Evidence-Based Medicine in Oncology: Commercial Versus Patient Benefit

**DOI:** 10.3390/biomedicines8080237

**Published:** 2020-07-23

**Authors:** Volker Schirrmacher, Tobias Sprenger, Wilfried Stuecker, Stefaan W. Van Gool

**Affiliations:** Immune-Oncological Center Cologne (IOZK), D-50674 Cologne, Germany; Sprenger@iozk.de (T.S.); stuecker@iozk.de (W.S.); vangoolstefaan@gmail.com (S.W.V.G.)

**Keywords:** randomized-controlled trial, evidence-based medicine, conflict of interest, adverse events, cancer immunology, immunotherapy, dendritic cell, oncolytic virus

## Abstract

At times of personalized and individualized medicine the concept of randomized- controlled clinical trials (RCTs) is being questioned. This review article explains principles of evidence-based medicine in oncology and shows an example of how evidence can be generated independently from RCTs. Personalized medicine involves molecular analysis of tumor properties and targeted therapy with small molecule inhibitors. Individualized medicine involves the whole patient (tumor and host) in the context of immunotherapy. The example is called Individualized Multimodal Immunotherapy (IMI). It is based on the individuality of immunological tumor–host interactions and on the concept of immunogenic tumor cell death (ICD) induced by an oncolytic virus. The evidence is generated by systematic data collection and analysis. The outcome is then shared with the scientific and medical community. The priority of big pharma studies is commercial benefit. Methods used to achieve this are described and have damaged the image of RCT studies in general. A critical discussion is recommended between all partners of the medical health system with regard to the conduct of RCTs by big pharma companies. Several clinics and institutions in Europe try to become more independent from pharma industry and to develop their own modern cancer therapeutics. Medical associations should include references to such studies from personalized and individualized medicine in their guidelines.

## 1. Introduction

Principles of evidence-based medicine became established in particular in the 1980s when many cytostatic drugs became approved. The practice combines personal clinical experience with the best external clinical evidence from systematic research [1]. The principles for external evidence are as follows: Once a drug is developed it has to be tested for safety and dose in a Phase I trial. Phase II trials aim to secure safety and to find out about effectivity. Phase III trials, executed with higher numbers of patients and performed prospectively and in a randomized-controlled way (i.e., randomized-controlled trials, RCTs), serve to establish effectivity in comparison to the control. Such studies are evaluated with the help of statistics to define probabilities and significances. A good example are the Kaplan–Meier curves for survival probability. If the therapeutic effect of a drug is low, the number of patients in a trial needs to be increased to achieve significance. This is the key for drug approval and entrance to the market.

This review compares large clinical studies performed mainly by big pharma companies with commercial interest to small clinical studies performed mainly by academic institutions for better treatment of cancer patients. As example for large studies serve RCTs for drug approval. As example for small studies serves individualized multimodal immunotherapy (IMI).

In view of the low effectivity of cytostatic drugs with regard to the most common human tumors, the carcinomas, basic and applied oncological research tried to explore entirely new pathways to achieve additional therapeutic effects against cancer. Molecular biologists developed targeted therapies (TTs) with small molecule inhibitors (SMIs). Virologists developed oncolytic viruses (OVs) and recombinant viral vectors with incorporated therapeutic transgenes. Immunologists developed monoclonal antibodies (mAbs), cancer vaccines and adoptive T cell therapies, including chimeric antigen-receptor (CAR) modified T cells.

The IOZK in Cologne (Germany) recently introduced a concept of Individualized Multimodal Immunotherapy (IMI) [2,3]. It involves a dendritic cell (DC) vaccine, IO-VAC^R^, an OV, Newcastle disease virus (NDV), and moderate electrohyperthermia (mEHT). The concept is based on the individuality of immunological tumor–host interactions and differs in many ways from the concept of RCTs. Side effects are low and innovative potential high. Based on systematic data collection and analysis, results have been published as single case reports or from case series studies. The IOZK is a small–medium independent enterprise and has no conflict of interest with big pharma companies.

This review is trying to compare the different worlds of RCT studies and those of IMI treatment.

## 2. Individuality of Immunological Tumor–Host Interactions

Traditionally, cancers are defined by histopathology. According to their tissue of origin, tumors are classified into four major groups: epithelial, mesenchymal, hematopoietic, and neuroectodermal. The most common human cancers, the carcinomas, are of epithelial origin. They are further subdivided into squamous cell carcinomas when they arise from epithelia that form protective cell layers and adenocarcinomas when they arise from secretory epithelia. Tissue sites of common types of adenocarcinoma are lung, colon, breast, pancreas, stomach, esophagus, prostate, endometrium, and ovary.

### 2.1. Tumor Properties

Recently, molecular biological and mass spectroscopic techniques, such as Next-Generation-Sequencing (NGS), has led to new insights into the biology of cancers. Genomics, epigenomics, transcriptomics, proteomics, peptidomics, ligandomics, and metabolomics (“OMICS” technologies) allow to uncover molecular features that distinguish cancer cells from healthy tissue [4]. Such techniques enabled the identification of key molecular pathways that control tumor progression. TTs with SMIs aim at blocking signal transduction through such pathways [5]. Imatinib mesylate (Gleevec), for example, the first successful SMI, functions as an antagonist of the Bcr-Abl tyrosine kinase active in CML and other leukemias [6].

OMICs and other technologies revealed that each cancer is unique in terms of genetics (mutations and others), epigenetics, antigenicity (tumor neoantigens), and immunogenicity [7,8].

### 2.2. Host Properties

Research from basic immunology enabled new insights into innate and adaptive anti-tumor immune reactivity. Investigation of metastasis formation and research of the tumor microenvironment (TME) led to further insights into the complexities of tumor–host interactions. Translational research from immunology provided new reagents for clinical application, for example mAbs, cytotoxic T lymphocytes (CTLs), interferons (IFNs), cytokines and chemokines. Clinical application of checkpoint inhibitory mAbs (e.g., anti CTLA-4 and anti-PD1) since 2011 has been a great success. They revealed that checkpoint receptors on T cells such as CTLA-4 and PD1 which mediate negative signals to T cells are being activated by tumors to escape immune destruction by CTLs.

The adaptive immunity system of humans, based on B and T lymphocytes, has a high diversity of antigen-recognition receptors, individually generated by somatic mechanisms. During ontogeny, the adaptive immunity system of humans learns to distinguish self (non-reactivity, tolerance) from non-self (reactivity). Tumor neoantigens are new (non-self) and not affected by tolerance mechanisms.

CD8+ CTLs and CD4+ T helper (Th) cells [9], in contrast to antibodies, have the ability to migrate through tissues and to infiltrate solid tumor tissue. The key anti-tumor effector cells are CTLs. They deliver a lethal hit signal towards target tumor cells and kill multiple tumor cells via the exocytosis of granzyme B containing cytotoxic granules [10]. In addition, Th cells locally produce cytokines in the tumor mass, creating a pro-inflammatory milieu and facilitating recruitment and activation of CTLs.

### 2.3. Tumor–Host Interactions

CTLs recognize tumor-associated antigens (TAAs). The first molecularly identified gene coding for a human TAA was an HLA-A1 restricted peptide that was not expressed in a panel of normal tissues. In the following decades, many more human TAAs were discovered. They all represented peptide-major histocompatibility (pMHC) molecule complexes. These findings revealed that T cells, in contrast to antibodies, can survey peptides created within cells. MHC molecules allow to transport peptides to the cell surface where they are presented to T cells. The molecular recognition of TAAs by T cells involves three participants: (i) an antigenic fragment (peptide, p) (ii) a presenter molecule (MHC) (it forms a complex with p and transports it to the cell surface) and (iii) a recognition molecule: the antigen-specific T cell receptor (TCR) [11].

MHC molecules are very polymorphic and their expression is highly individual. The pathways of MHC class I and MHC class II antigen presentation ensure that most of the cells of the body are permanently screened for the presence of altered peptides. Every tumor may contain a few hundreds of mutations in coding regions of the genome. In addition, deletions, amplifications, and chromosomal rearrangements can result in new genetic sequences. The vast majority of mutations occur in intracellular proteins [12]. The mutational burden is highly correlated with the neo-epitope load [8].

Antigen-specific TCRs are being generated during ontogeny within the thymus. Their diversity, individually generated by somatic mechanisms, is very high. Only mature T cells with receptors that do not react with “self” pMHC complexes are allowed to leave the thymus (central tolerance mechanism [13]). Their receptors have the capacity to react against “non-self” peptides. Non-self peptides generated by tumors through the above mentioned mechanisms are individually unique and called tumor neoantigens [7]. They are particularly important for tumor rejection because they have not been affected by central tolerance mechanisms. T cells recognize tumor neoantigens as peptides displayed by MHC molecules on the surface of antigen-presenting cells such as dendritic cells (DCs).

Even if tumors express tumor neoantigens, this is not sufficient for immunogenicity, i.e., their capacity to generate an immune response. Immunogenicity requires T cell costimulatory and danger signals [14]. CD28 is the principal costimulatory receptor for delivering second signals for T cell activation. These molecules bind to B7 (CD80, CD86) costimulatory molecules expressed by professional antigen-presenting cells, such as DCs. CD4+ Th cells can provide the necessary costimulatory signals to CD8+ CTLs [9]. Also, signal 3 from innate immunity cells (IL-12 or type I interferon), often associated with microbial infections and danger, can provide effective CTL survival and improvement of effector function [15]. As recently discovered, T cell costimulation in anticancer immunity has metabolic consequences [16,17]. Such metabolic activities allow fully activated T cells to exert their many functions, such as proliferation, differentiation, migration in tissues, and exertion of anti-tumor effector functions.

The main points contributing to the individuality of tumor–host interactions can be summarized as follows: 1. Each cancer is unique. 2. The high diversity of B and T cell receptors is generated individually by somatic mechanisms. 3. Tumor neoantigen pMHC complexes are individually unique.

A fourth point concerns cancer-reactive memory T cells (MTCs) [18] which maintain once the T cell effector phase is terminated. Cancer-reactive MTCs can be generated by active-specific immunization with cancer vaccines. They can also be generated spontaneously in patients [19]. The repertoire of their specificities, as analyzed from bone marrow samples, is highly individual [19].

“Innate and adaptive immunity in the tumor microenvironment” is the title of a book recommended for further reading including 10 chapters and a number of clarifying pictures [20].

In conclusion, T cells are of major importance for tumor immune surveillance. They can screen peptides from intracellular proteins and they can infiltrate into solid tumor masses. That is why tumors often develop immune escape mechanisms [21] to shut down host anti-tumor T cell reactivity. The application of neutralizing antibodies against checkpoint inhibitors revitalizes a suppressed immune system [22]. The clinical effects reveal the extent and importance of tumor–host immune cell interactions over long time periods in cancer patients.

## 3. Individualized Multimodal Immunotherapy (IMI) and Patient Benefit

### 3.1. Concept

The objective of IMI developed by IOZK [2,3] is to stimulate strong specific T cell mediated immune responses against TAAs in cancer patients, in particular against unique tumor neoantigens [7]. A cancer vaccine called IO-VAC^R^ is produced individually for each patient. Tumor material and antigen-presenting DCs of the vaccine are strictly autologous. The third component of IO-VAC^R^ is an avian OV, namely NDV. This serves to augment the immunogenicity of the vaccine. The virus has been successfully produced by IOZK in 2015, the first time worldwide, according to high quality standard Good Manufacturing Practice (GMP). IO-VAC^R^ is an approved Advanced Therapeutic Medicinal Product (ATMP) in North Rhine-Westphalia, Germany, and is applied to patients at IOZK on a compassionate use basis [3].

Active-specific immunization with IO-VAC^R^ is only one modality of the multimodal concept (Table 1). The second modality is pre-treatment of the patient with oncolytic NDV to induce in situ immunogenic tumor cell death (ICD) [3]. This pre-treatment is combined with modality 3, local moderate electrohyperthermia (mEHT). The rationale of the second and third modality is immune modulation/conditioning of the patient‘s immune system one week before vaccination to achieve the best possible effects of vaccination. The patient’s immune system should not be dysregulated but rather polarized towards a Th1 cellular immune response [9]. Details of the scientific background, the production and the application protocol have been described [2,3].

**Figure 1:**
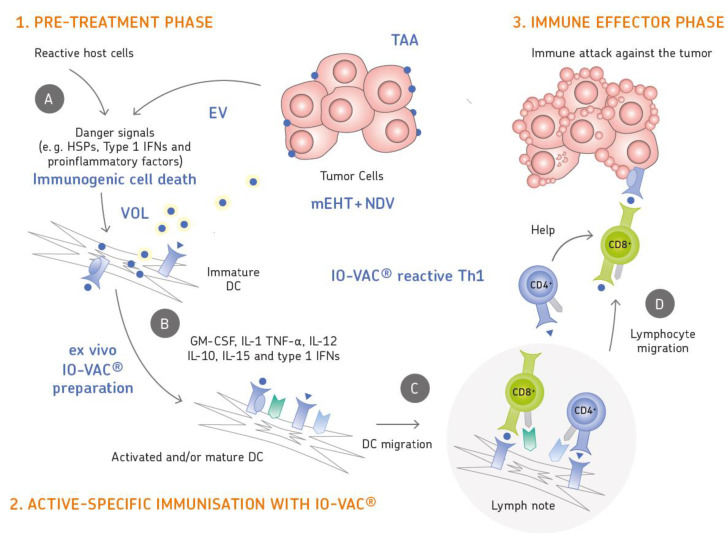
Cartoon of the three phases of Individualized Multimodal Immunotherapy (IMI) (**1**–**3**.) and of respective tumor–host interactions (**A**–**D**). **1**–**3**: 1. The pre-treatment phase involves 5 sessions of moderate electrohyperthermia (mEHT) and Newcastle disease virus (NDV) treatment within the first week. This leads to ICD and to release of extracellular vesicles (EV) which initiate anti-tumor immune responses. 2. Active-specific immunization at day 8 with IO-VAC^R^ represents the second phase of treatment. It involves intradermal application of fully matured tumor-associated antigen (TAA) presenting DCs. 3. In the immune effector phase, about 1 to 5 days after vaccination, the activated cancer-reactive T cells leave the lymph node, migrate to the tumor and attack tumor cells by TAA-specific cytotoxic T lymphocytes (CTLs). This causes their destruction as visualized by the blebs. (**A**–**D**): (**A**) illustrates immunogenic cell death with the release of viral oncolysate (VOL) from tumor cells treated by mEHT and NDV. (**B**) shows the process of loading dendritic cells ex vivo with TAAs and DC maturation via growth factors, cytokines and interferons to produce the vaccine IO-VAC^R^. (**C**) illustrates DC migration to local lymph nodes and their cognate interaction with TAA-specific CD4 Th1 cells and CD8 CTLs. (**D**) shows migration of activated T lymphocytes out of the lymph node and trafficking via the blood towards the site of the tumor. For abbreviations see alphabetic list at the end. The figure was designed by Riegel and Reichenthaler.

Figure 1 illustrates the three phases of IMI: 1. Pre-treatment, 2. Active-specific immunization, 3. Immune effector phase. These phases involve various kinds of tumor–host interactions. A illustrates immunogenic cell death with the release of viral oncolysate (VOL) from tumor cells treated by mEHT and NDV. B shows the process of loading dendritic cells with TAAs and DC maturation. C illustrates DC migration to local lymph nodes and their cognate interaction with TAA-specific CD4 Th1 and CD8 CTLs. D shows migration of activated T lymphocytes out of the lymph node and trafficking via the blood towards the site of the tumor. The effector phase causes tumor cell destruction.

One cycle of IMI takes 8 days for the patient on an ambulant basis. Usually, two cycles of IMI suffice for the whole treatment process. The aim is not only the specific destruction of tumor cells but also the induction of cancer-reactive memory T cells.

Table 1 contains the main features of IMI.

### 3.2. Safety Aspects

At times of the pandemic COVID-19, it may be appropriate to mention that RNA viruses like corona are highly susceptible to UV-B light. NDV is a negative-stranded RNA virus of birds (permissive host species). It is completely inactivated by a short exposure to UV-B light. NDV has a high safety profile in man. More than 50 years of clinical application witness this [29].

Special safety characteristics of NDV include: (i) Lack of gene exchange via recombination, (ii) lack of interaction with host cell DNA, (iii) independence from host cell proliferation (the virus replicates in the cell’s cytoplasm), (iv) selectivity of virus replication and cell lysis (oncolysis) for tumor cells in non-permissive hosts such as human. Reasons for the tumor selectivity of NDV have been described in detail [25,29].

### 3.3. Clinical Results

Results from IMI treatment of cancer patients at IOZK have been published as either single case reports or as case series studies.

One case concerns a breast cancer patient with extensive liver metastases who was operated but refused further standard therapy. After IMI treatment a continuous high quality of life was reported. The metastases apparently became encapsulated. No further metastases developed and the patient survived more than 66 months after the initial diagnosis. The patient developed a long-lasting tumor-reactive memory T cell response [26].

Another case report concerns long-term remission of prostate cancer with extensive bone metastases [27]. The patient had received standard therapy but this had failed. After IMI, the patient achieved complete remission. The patient also developed a long-lasting anti-tumor memory T cell response.

A case series report relates to adult patients with glioblastoma multiforme (GBM) (*n* = 34). The patients received standard radiochemotherapy in combination with IMI. Median overall survival (OS) was 23.4 months [3,28]. Radiochemotherapy alone according to the Stupp protocol had been reported to achieve a median OS of 14.6 months.

Another case series report relates to children with diffuse intrinsic pontine glioma (DIPG) (*n* = 41). The prognosis of children with DIPG remains very bad even after radio- and chemotherapy or after molecular-targeted therapy. IMI was feasible without major toxicity and median OS appeared longer than expected from a retrospective analysis. A longer OS was associated with a Th1 shift [28]. This T cell polarized response is in line with the concept of IMI [2,3].

### 3.4. Innovation

#### 3.4.1. Immunogenic Cell Death (ICD) Induction Therapy and Liquid Biopsy

The vaccine IO-VAC^R^ is normally produced by loading autologous DCs with oncolysate from tumor cells derived from surgical resectates and thereof cultured cells. In many cases tumor specimens are not available due to inoperability or because the tumor had already been removed before considering immunotherapy. Therefore, IOZK is developing a technique of isolating tumor material from serum of patients. These are treated for five consecutive days with intravenous injection of oncolytic NDV in combination with mEHT via the Oncothermia EHY-2000 device (Oncotherm GmbH, Troisdorf, Germany). A new PanTum Detect assay evaluates by FACS analysis peripheral blood derived phagocytic monocytes/macrophages with ICD derived tumor markers using the Epitope Detection in Monocytes (EDIM) technology [28]. It revealed that during these five days of treatment ICD-induced tumor products are increasingly detectable in the serum. These are antigenic extracellular microvesicles and apoptotic bodies. Such serum material was used in case of the non-operable children with DIPG tumors to load their DCs [28].

Also, plasma circulating tumor DNA (ctDNA) analysis was used to screen for distinct mutations in 21 DIPG patients. Besides, circulating tumor cells (CTC) were isolated and the mRNA expression level of PDL1 was analyzed by Biofocus (www.biofocus.de).

These procedures of Liquid Biopsy are just one example of innovative technologies performed at IOZK in addition to IMI [28].

#### 3.4.2. Potential to Break Therapy Resistance

Resistance to therapy is a major obstacle to cancer treatment. There are a number of anti-neoplastic effects of NDV in non-permissive hosts like cancer patients [29]. Some properties appear to have the potential to break therapy resistance of various kinds.

First, there are special features that explain tumor selectivity of NDV [30]: (i) Targeting the oncogenic protein Rac1, (ii) tumor-selective virus replication, (iii) promotion of virus propagation via syncytia, autophagy, and exosomes, (iv) tumor-selective oncolysis and (v) tumor-selective induction of immunogenic cell death.

Secondly, there are a variety of features of NDV which are of relevance for resistance to therapy: (i) Potential to break T cell tolerance towards TAA expressing tumor cells, (ii) potential to break resistance to chemotherapy or radiotherapy, (iii) potential to break resistance to apoptosis, (iv) potential to break resistance to hypoxia, (v) potential to break resistance to TNF-related apoptosis-inducing ligand (TRAIL), (vi) potential to break resistance to immune checkpoint blockade, and (vii) potential to break resistance to anti-viral immunity [24,25].

Any one of these features of NDV could have contributed to the positive case reports of patients with metastasized breast cancer [26] and metastasized prostate cancer previously resistant to standard therapy [27].

#### 3.4.3. Side Effects, Benefit for the Patient, Affecting the Tumor Microenvironment, and Improvement of Knowledge

The IMI at IOZK involving cancer vaccines and oncolytic virus treatment exerts profoundly lower side effects than other systemic therapies [23]. Cancer treatments by means of cytostatic drugs, SMIs, checkpoint inhibitory antibodies or CAR-T cells can produce major adverse events (AEs) of grades 3–4 (Section 5).

The side effects of treatments at IOZK are well tolerated (grades 1–2). A main reason is that application of vaccines and OVs elicit physiological processes [23].

All of the procedures of treatment at IOZK and their possible manifold multimodal combinations aim at an optimal individual immunological management of a cancer patient.

The TME is a tumor surrounding tissue constructed by manifold tumor–host interactions [20]. The tumor uses it to support its own growth, e.g., via angiogenesis, and to protect itself against attacks by the host’s immune system. Apart from silencing CTLs, the TME attracts immune suppressive cells, e.g., myeloid-derived suppressor cells (MDSCs), M2 macrophages and T regulatory (Treg) cells. The treatment strategy of IOZK includes application of inhibitors of immune suppressive cells and also application of checkpoint inhibitory antibodies, if required.

Systematic data collection and analysis allows continuous learning. Publication of the results [2,3,23,24,25,26,27,28,30] ensure transparency for the scientific and medical community.

## 4. Comparison between Randomized-Controlled Trials (RCTs) for Drug Approval and IMI

The two examples of clinical studies with high or low number of patients are now being compared. Table 2 includes 13 features of RCTs and IMI.

1. and 2. GMP is a high quality production prerequisite for any new drug on the market, including RCTs and IMI. The production rate of a drug is high for RCTs and low (just one; individually produced vaccine) for IMI.

3. The number of patients per trial. The above mentioned case series studies from IOZK involved 34 and 41 patients. In contrast, RCT studies normally involve >1000 patients.

4. Individual patient benefit: The recruitment of patients into an RCT is not easy and pharma companies offer money per patient to participating clinics. Many patients want to get a new treatment rather than to get randomized. There is no individual therapy benefit for patients in the control arm. RCTs determine the mean treatment effect and provide a general statistical estimate of efficacy for the study population under investigation [1]. RCTs have only limited significance for the single patient [31]. For IMI every patient gets the same chance of benefit.

5. LOE: The Oxford Center for Evidence-Based Medicine has developed a schema of “Level of Evidence” (LOE) to which medical guidelines usually refer. Systematic reviews of RCTs, meta-analyses, are regarded as the highest level (LOE 1a). LOE 5 means that scientific evidence is inherently acknowledged for innovations derived from basic research and long-standing medical experience with novel clinical applications, such as IMI.

6. Number of variables per study: In a two-arm RCT study, the number of variables that can be tested is very low. In contrast, in IMI there are many possible variables due to the multimodality concept.

7. Risk of resistance development: Many cancer therapies have to face the problem of therapy resistance development. This is true not only for cytostatic drugs but also for new drugs including SMIs and also for tumor antigen targeted immunotherapies. There are two features of IMI that reduce the risk of resistance development: (i) The inclusion of oncolytic NDV with its potential to break therapy resistance (see Table 1) and (ii) the multimodality approach including physical (hyperthermia) and biological treatment procedures.

8. and 9. Flexibility is another important parameter. The first approved therapeutic cancer vaccine may serve as an example. Sipuleucel-T was approved in 2010 for metastatic prostate cancer. It is a DC-like vaccine pulsed with recombinant fusion protein composed of prostatic acid phosphatase (PAP) fused to granulocyte macrophage colony-stimulating factor (GM-CSF). At the time of approval the technology for production of DC based vaccines was still relatively poor. Adaptations to state-of-the-art production is not possible after drug approval. Another problem with sophisticated technology are the costs. Unfortunately, the producer (Dendreon Co., Seattle, WA, USA) filed for bankruptcy in 2014. Thus, methodological innovation after drug approval by RCTs is low. With IMI, adaptations to state-of-the-art are possible and the potential for methodological innovation is high.

10. Costs: With greater than 20 million $, the costs for executing an RCT are very high. The costs of newly approved cancer drugs are up to >100,000 $ per year. This is not in any reasonable relation to their often modest additional benefits [31]. High prices exclude independent comparative efficacy trials aimed at establishing equivalent but cheaper alternatives. High drug prices thus protect the market share of expensive drugs [32,33].

Neither small-medium independent research institutions nor public institutions can afford this type of RCT studies. Independent, innovative, principal investigator (PI) driven studies are virtually impossible. As a consequence, big pharma companies dominate the drug market, both in the US and in Europe. Only they can afford Phase II and III studies that last long and costs a lot of money. The developmental costs are afterwards passed on to the consumer.

11. Price per drug. The price for IMI treatment at IOZK is about 55,000 Euro and is based on the legally fixed prices for ambulant treatment in Germany. This has to be payed privately or it is covered by health insurances. The price just covers the enormous investments for GMP production by this private non-profit enterprise. This situation is in sharp contrast to approved drugs that are put onto the market by big pharma institutions. Their drugs are not produced individually and have a high production rate for worldwide consumption. Nevertheless, the price is by far higher than that of IMI. A commercial benefit for them is evident from this comparison.

12. and 13. Primary aim and conflict of interest: The primary aim of RCTs is drug approval and thus a commercial interest. IMI studies, in contrast, aim at a benefit for the cancer patient. Because of this difference in primary aim, a conflict of interest is high in connection with RCTs and low with IMI.

## 5. RCTs for Drug Approval and Commercial Benefit

Evidence-based medicine is important for medical progress. For a long time RCTs have been considered as gold standard for establishing new therapies in oncology. With time, RCTs in oncology have become larger and their funding shifted from government (1975–1994: 60%) to industry (1995–2004: 57%) [34]. An analysis from 2008 of 321 eligible RCTs revealed a statistically significant association between for-profit sponsorship and the reporting of positive results with endorsement of the experimental arm [34].

The role of RCTs is more and more debated and questioned [35]. Scientists criticize that a majority of medical doctors and decision makers are not able to safely assess the credibility and benefit of medical evidence from RCTs. They complain about a medical misinformation mess [36]. Better designed and more rigorous RCTs are needed to develop an evidence base that can decisively provide reliable effect estimates.

The power and attitude of big pharma companies in the last decades has set priorities in commercial rather than patient benefit. To support this statement, Table 3 summarizes a variety of features such as fragility index, survival, quality of life, side effects and bias. These features have been evaluated with respect to RCTs by meta-analysis, the highest level of evidence.

1. The fragility index [37] is a statistical measure to evaluate the reliability of study results. The evaluation of new cancer drugs approved by the FDA between 2014 and 2018 revealed a low fragility index and thus a low reliability. The majority of RCTs provided no clinically relevant benefit [44,45,46].

2. Benefit in overall survival (OS) is considered of high relevance for cancer patients. According to an evaluation of new drugs approved by the EMA between 2009 and 2013 [38], most drugs had no benefit in OS. To accelerate drug approval, many companies investigated surrogate parameters instead of OS. This applied to 84% of RCTs in Europe [38].

3. The same evaluation study revealed that the new cancer drugs approved by EMA had no benefit for Quality of Life (QoL) [38].

4. Toxicity: A meta-analysis of newly FDA approved drugs revealed not only high costs [39] but also high risks of associated toxicities [40]. The NCI Common Terminology Criteria for Adverse Events v4.0 (CTCAE) from 2009 defines 5 grades of severity of adverse events (AEs). A low intensity scale reaches from mild (1), to moderate (2). A high intensity scale starts with severe (3) and goes via life-threatening (4) to death related to AE (5) [23]. Grade 3 and 4 toxicities include, among others, in alphabetic order, blood and lymphatic system disorders, cardiac disorders, endocrine disorders, gastrointestinal disorders, hepatobiliary disorders, musculoskeletal and connective tissue disorders, nervous system disorders, psychiatric disorders, renal and urinary disorders, respiratory, thoracic and mediastinal disorders and skin and subcutaneous tissue disorders. Neurotoxic effects of grade 4 include spasms, paralysis and coma [23]. Pharmaceutic companies and oncologists are familiar with such toxic effects from cytostatic drugs.

5. NNT: A cancer patient does not want to suffer toxic side effects without benefit. But this is exactly the case with drugs approved from RCTs which measure a number to treat (NNT) to avoid an additional event (tumor progression or death). Here is an example from endometrial carcinoma. After hysterectomy and radiotherapy, a statistically significant survival advantage was confirmed for additional chemotherapy [41]. It may be that 33 patients have to receive a potentially toxic therapy in order that one of them has a benefit. This means that a majority of 32 patients receive a potentially toxic therapy without benefit.

6. Bias: The evaluation of new cancer drugs approved by EMA between 2014 and 2016 revealed that about 50% of the approved studies were classified as highly biased. The side effects were not properly recorded, there were also other methodological shortcomings and an overestimation of benefit [42].

The risk-of-bias assessment should be based on the hierarchical ranking order of the WHO linked to health condition: (i) Mortality, (ii) morbidity (treatment failure, pathology; symptoms of disease), (iii) health impairment (loss/abnormality of function, incl. presence of pain), (iv) limitation of activity (disability, incl. days off work/school because of ill health), (v) restriction of participation (quality of life), (vi) surrogate outcome (e.g., blood test data, bone mineral density) [47].

7. Guidelines biased by conflict of interest: Guidelines are frequently used as a reference for health insurance companies. They can be deformed by economic interests and fundamental conceptual limitations. The results from a review of the oncology guidelines published by the National Comprehensive Cancer Network are shown at the bottom of Table 3. The content of conventional guidelines is dominated by a few “key opinion leaders” who are likely to have conflicts of interest [43].

## 6. Personalized and Individualized Medicine Requires New Types of Clinical Studies

The remarkable increase in knowledge in molecular biology, virology and immunology and the many new concepts of translational research call for changes in the methodology of clinical trials [48,49]. For instance, it is recommended to establish databases of results from individual patients treated with personalized or individualized medicine for early identification of effective drugs or treatment strategies in a larger patient population [50]. Basket trials allow a drug to be tested simultaneously in subgroups of different tumor types [51]. Umbrella trials enable the evaluation of several targeted therapies for a single disease [51]. It has been suggested that it is time for one-person trials [52]. In N-of-1 trials, the acquisition of knowledge is coupled with an individual benefit for the single patient. Such a study design could lead to a significant reduction in costs [53].

IMI is only one example for small-type clinical studies. Other examples are post-operative active-specific immunotherapy studies with the autologous virus-modified vaccine ATV-NDV. Such studies were performed, statistically evaluated and published for breast cancer [54], colon cancer [55], GBM [56], and Head and Neck Squamous Cell carcinoma [57]. One of these small clinical studies was performed as RCT [58]. It revealed for colon cancer patients scientific validity by demonstrating an extremely high efficacy although the number of patients was small. Another example is a case-series study with oncolytic virotherapy, recently published [59]. It revealed effective treatment of four GBM patients who achieved clinical and radiological responses with long-term survival and good quality of life.

Considering the deficits of RCTs, it is time that medical organizations and health insurances become aware of this. They should become more independent from big pharma studies and support new types of clinical studies.

The development of CAR-T cell therapy in Europe is an example of how university clinics try to become independent from big pharma companies. According to a recent report, clinics in Germany, Switzerland, Italy, and Spain develop their own CAR-T cell products. These will be continuously updated and will be cheaper than the big pharma products [60].

## 7. Discussion

The speed of approval of new drugs by regulatory agents such as FDA and EMA is increasing since years. In clinical practice, the additional benefit of the new drugs is questioned. Oncologists lack full information of relevant details such as real extent of effects and side effects. There is a trend to consider RCTs as obsolete for the daily clinical practice.

The fact that FDA and EMA receive large amounts of money from pharma companies question the independence of these agencies [61]. The consequence of this development is particularly problematic for cancer patients. They have no chance to judge about the usefulness and side effects of a tremendous number of new drugs. Even oncologists cannot tell the difference, for example, between MK-2206, GSK690693 and AZD8055 (all SMIs: PI3K-AKT-mTor inhibitors) or between Perjeta, Cyramza and Darzalex (all mAbs: against HER2, VEGFR2 and CD38). Even after introduction of generic names, it is not easy to distinguish, for example, between buparlisib, pictisilib, and idelalisib (all SMIs: PI3-AKT-mTor inhibitors) and pertuzumab, ramucirumab, and daratumumab (all mAbs: against HER2, VEGFR2, and CD38).

In contrast to government funded or PI initiated clinical studies, for big pharma sponsored RCTs commercial benefit comes first. Apparently, deception and deliberate tricks are being used to obtain a commercial benefit. A book analyzing conflicts of interest in medicine comprises 19 chapters and more than 300 pages [61].

Meanwhile, rules of evidence based medicine, such as meta-analyses, provide evidence for the kind of business of big pharma companies. The evidence summarized in Table 3 reveals the following: The quality of a majority of approved drugs is too low and the prices too high. The public health system is an important market for large pharma companies. Therefore they invest a lot of money for drug promotion [62]. The consequences for the cancer patient are severe: Low effectivity and high toxicity (AEs of Grade 3 and 4). In the interest of cancer patients, oncologists should be very critical to influences via pharma lobbyists.

Considering decades of development, much progress has been made in anti-cancer drugs. For example: Molecular targeted drugs such as SMIs. In comparison to cytostatic drugs, SMIs target pre-defined subsets of patients and thereby achieve a higher effectivity. Major innovatory therapeutics derived from basic research in immunology: checkpoint inhibitory mAbs, DCs, and cancer vaccines, oncolytic viruses or CAR-T cells. It were always scientists who introduced an innovation first and then (not always) big pharma companies became involved. Principles for production of mAbs were developed by the Nobel Laureates from 1984 NK Jerne, GJF Köhler and C Milstein. DCs and their culture technology were developed by the Nobel Laureate from 2011, RM Steinman. Immune checkpoint therapy was developed by the Nobel Laureates from 2015 JP Allison and T Honjo. The discoveries of DCs by RM Steinman and of Toll-like receptors (TLRs) for innate immunity activation by the Nobel Laureates from 2011 B Beutler and J Hoffmann also influenced the development of the concept of IMI at IOZK, Cologne, Germany.

To reduce side effects (AEs of Grade 3 to 4) from systemic cancer treatment is an important goal to improve cancer therapy. IMI developed by IOZK provides an example by using physiological types of therapy such as cancer vaccines and oncolytic viruses [23]. A position paper [63] of IOZK underlines the reasons why clinical studies need to be adapted to the new insights into the immunobiology and dynamics of tumor–host interactions [63].

## 8. Conclusions

Evidence-based medicine is important for progress in oncology, including patient safety and high-quality treatment. The question is how to generate appropriate evidence. The highest level of evidence is traditionally assigned to randomized-controlled clinical trials. There are two developments which challenge this position: (i) Personalized medicine with “OMICs” technologies and individualized medicine with innovative immunotherapeutic ideas and (ii) non-correct conduct of RCTs by big pharma companies. The first development is for the patient’s benefit, the second for commercial benefit.

Conflicts of interest exist not only in the approval of oncological pharmaceuticals but also in the development of guidelines. RCTs form the basis for the development of guidelines and this has consequences for clinical decisions and for reimbursement by statutory and private health insurance companies. RCTs thus have far reaching effects within the private and public health sector.

Spending on oncologic pharmaceuticals keeps rising in terms of costs for private and public health systems worldwide with minimal clinical advances. This is due to RCTs, thereof derived fragile data and conflicts of interest. Meta-analyses, a procedure of evidence-based medicine, provide evidence that in the last decade a majority of approved drugs from RCTs have: (i) Low reliability; (ii) no benefit in OS or QoL; (iii) high costs; and (iv) a high risk of associated severe toxicities.

Drug approval by regulatory authorities apparently does not consider AEs of Grade 3 and 4 sufficiently important for study rejection. Such strong AEs cause additional sufferings for cancer patients and prevent a beneficial effect.

Cancer vaccines and oncolytic viruses are well tolerated with side effects below grade 2. Such advanced therapeutic medicinal products (ATMPs) are part of an individualized multimodal immunotherapy (IMI). Systematic data collection and analysis of single cases or case series allows to generate new medical data and evidence, thus leading to progress in oncology. Cancer vaccines and oncolytic viruses have the potential to stimulate the immune system against cancer and to establish cancer-reactive immunological memory. This is important for patient welfare, quality of life, and quality of survival (QoL/QoS). Such evidence and treatments for patient benefit should be taken more seriously by medical associations and health insurance companies.

Progress in oncology depends on better treatment of cancer patients. Personalized and individualized medicine contribute to such progress. The reduction of side effects by introduction of physiologically oriented treatment concepts is another important aspect. Big pharma conducted RCTs with their interest in commercial benefit contribute to increase of costs but comparatively less to better treatment of patients. A medical health system/pharma network-critical discussion is recommended to improve the treatment of cancer patients and to save money within the system.

## Figures and Tables

**Table 1:** Individualized Multimodal Immunotherapy (IMI).

Feature	Example IOZK			Reference
Concept	Modality 1	Active-specific immunization	IO-VAC^R^ (vaccine)	[2]
	Modality 2	Treatment with oncolytic virus	NDV ^1^ (OV)	[3]
	Modality 3	Combination with mEHT ^2^	EHY-2000 (Oncothermia)	[2,3]
Side effects	low	WHO Grade	<2	[23]
Innovation	In situ induction of ICD ^3^	Potential to break therapy resistance		[3,24,25]
Single cases	Breast cancer	Prostate carcinoma		[26,27]
Case series	GBM ^4^ adults	DIPG ^5^ children		[3,28]

^1^ NDV = Newcastle disease virus; ^2^ mEHT = moderate electrohyperthermia; ^3^ ICD = Immunogenic cell death; ^4^ GBM = Glioblastoma multiforme; ^5^ DIPG = Diffuse intrinsic pontine glioma.

**Table 2:** Comparison between randomized-controlled trials (RCT) and Individualized Multimodal Immunotherapy (IMI).

Feature	RCT ^1^ for Drug Approval	IMI ^2^
1. GMP ^3^ quality standard	Yes	Yes
2. Production rate of drug	High	Low
3. Number of patients per trial	>1000	<50
4. Individual patient benefit	Low	High
	No benefit in control arm	Same chance for every patient
5. LOE ^4^	1	5
6. Number of variables included	Only few possible	Many possible
7. Risk of resistance development	High	Low
8. Flexibility: Adaptations to state of the art	Not possible after approval	Possible
9. Methodological innovation	Low	High
10. Costs	Very high (>20 million $)	Comparatively low
11. Price per drug	Very high	Comparatively low
12. Primary aim	Commercial (Drug approval)	Patient benefit
13. Conflict of interest	High	Low

^1^ RCT = Randomized controlled trial; ^2^ IMI = Individualized multimodal immunotherapy; ^3^ GMP = Good manufacturing practice; ^4^ LOE = Level of evidence.

**Table 3:** Randomized-controlled clinical trials (RCTs) for drug approval and commercial benefit.

Feature	Results	Conclusion	Comment	Reference
1. Fragility index ^1^	<50% of RCTs with clinically relevant benefit ^2^	Low (<2)		[37]
2. Survival (OS ^3^)	84% of RCTs investigated surrogate parameters ^4^	Most drugs had no benefit in OS		[38]
3. Quality of Life		No benefit ^4^		[38]
4. Toxicity		High risk of associated toxicities		[39,40]
5. NNT ^5^		A relatively high number of patients to be treated to avoid one additional event		[41]
6. Bias	87% of authors received payment from industry	About 50% of approved studies were classified as highly biased ^4^		[42]
7. Oncology guidelines	72%of recommendations based on weak evidence	Dominated by a few key opinion leaders with conflict of interest	Often used as reference for reimbursement	[43]

^1^ Fragility index = a statistical measure to evaluate the reliability of study results. A factor of 1 means that study results are fragile and not reliable; ^2^ = Evaluation of new cancer drugs approved by the FDA (2014–2018); ^3^ OS = Overall Survival; ^4^ = Evaluation of new cancer drugs approved by the EMA (2009–2013); ^5^ NNT = Number to treat.

## Abbreviations

AE: Adverse Event; ATMP: Advanced Therapeutic Medicinal Product; ATV-NDV: Virus modified Autologous Tumor Cell Vaccine; CAR: Chimeric Antigen Receptor; ctDNA: circulating tumor DNA; CTL: Cytotoxic T Lymphocyte; DC: Dendritic Cell; DIPG: Diffuse Intrinsic Pontine Glioma; EDIM: Epitope Detection in Monocytes; EV: Extracellular Vesicle; GBM: Glioblastoma Multiforme; GM-CSF: Granulocyte-Macrophage Colony Stimulating Factor; GMP: Good Manufacturing Praxis; HLA: Human Leucocyte Antigen; HSP: Heat Shock Protein; ICD: Immunogenic Cell Death; IL(-1, -10, -12, -15): Interleukins; IFN: Interferon; IMI: Individualized Multimodal Immunotherapy; IOZK: Immune-Oncological Center Cologne; LOE: Level of Evidence; MDSC: Myeloid-Derived Suppressor Cell; mEHT: moderate Electrohyperthermia; MHC: Major Histocompatibility Complex; MTC: Memory T Cell; NDV: Newcastle Disease Virus; NGS: Next-Generation Sequencing; NNT: Number to Treat; OV: Oncolytic Virus; OS: Overall Survival; PAP: Prostatic Acid Phosphatase; PI: Principal Investigator; pMHC: Peptide-MHC Complex; QoL: Quality of Life; QoS: Quality of Survival; RCT: Randomized Controlled Trial; SMI: Small Molecule Inhibitor; TAA: Tumor Associated Antigen; TCR: T Cell Receptor; Th cell: T helper cell; TME: Tumor Microenvironment; TNFα: Tumor Necrosis Factor alpha; TRAIL: TNF-Related Apoptosis Inducing Ligand; TT: Targeted Therapy; VOL: Viral Oncolysate; WHO: World Health Organization

## References

[1] Sacket D.J., Rosenberg W.M., Gray J.A., Haynes R.B., Richardson W.S. (1996). Evidence-based medicine: What is it and what it isn’t. BMJ.

[2] Schirrmacher V., Lorenzen D., Van Gool S.W., Stuecker W. (2017). A new strategy of cancer immunotherapy combining hyperthermia/oncolytic virus pretreatment with specific autologous anti-tumor vaccination-A review. Austin Oncol. Case Rep..

[3] Van Gool S.W., Makalowski J., Feyen O., Prix L., Schirrmacher V., Stuecker W. (2018). The induction of immunogenic cell death (ICD) during maintenance chemotherapy and subsequent multimudal immunotherapy for glioblastoma (GBM). Austin Oncol. Case Rep..

[4] Chakraborty S., Hosen M.I., Ahmed M., Shekhar H.U. (2018). Onco-Multi-OMICS Approach: A new frontier in cancer research. Biomed. Res. Int..

[5] Krzyszczyk P., Acevedo A., Davidoff E.J., Timmins L.M., Marrero-Berrios I., Patel M., White C., Lowe C., Sherba J.J., Hartmanshenn C. (2018). The growing role of precision and personalized medicine for cancer treatment. Technology.

[6] Savage D.G., Antman K.H. (2002). Imatinib mesylate-A new oral targeted therapy. N. Engl. J. Med..

[7] Gubin M.M., Artyomov M.N., Mardis E.R., Schreiber R.D. (2015). Tumor neoantigens: Building a framework for personalized cancer immunotherapy. J. Clin. Investig..

[8] Narang P., Chen M., Shama A.A., Anderson K.S., Wilson M.A. (2019). The neoepitope landscape of breast cancer: Implications for immunotherapy. BMC Cancer.

[9] Bevan M.J. (2004). Helping the CD8+ T cell response. Nat. Rev. Immunol..

[10] Chang H.F., Bzeih H., Schirra C., Chitirala P., Halimani M., Cordat E., Krause E., Rettig J., Pattu V. (2016). Endocytosis of cytotoxic granules is essential for multiple killing of target cells by T lymphocytes. J. Immunol..

[11] Rossjohn J., Gras S., Miles J.J., Turner S.J., Godfrey D.I., McCluskey J. (2015). T cell antigen receptor recognition of antigen-presenting molecules. Annu. Rev. Immunol..

[12] Nik-Zainal S., Davies H., Staaf J., Ramakrishna M., Glodzik D., Zou X., Martincorena I., Alexandrov L.B., Martin S., Wedge D.C. (2016). Landscape of somatic mutations in 560 breast cancer whole genome sequences. Nature.

[13] Kurd N., Robey E.A. (2016). T-cell selection in the thymus: A spatial and temporal perspective. Immunol. Rev..

[14] Baxter A.G., Hodgkin P.D. (2002). Activation rules: The two-signal theory of immune activation. Nat. Rev. Immunol..

[15] Curtsinger J.M., Gerner M.Y., Lins D.C., Mescher M.F. (2007). Signal 3 availability limits the CD8 T cell response to a solid tumor. J. Immunol..

[16] MacIver N.J., Michalek R.D., Rathmell J.C. (2013). Metabolic regulation of T lymphocytes. Annu. Rev. Immunol..

[17] Teijeiras A., Garasa S., Etxeberria I., Gato-Canas M., Melero I., Delgosse G.M. (2019). Metabolic consequences of T-cell costimulation in anticancer immunity. Cancer Immunol. Res..

[18] Lanzavecchia A., Sallusto F. (2000). From synapses to immunological memory: The role of sustained T cell stimulation. Curr. Opin. Immunol..

[19] Sommerfeld N., Schütz F., Sohn C., Förster J., Schirrmacher V., Beckhove P. (2006). The shaping of a polyvalent and highly individual T-cell repertoire in the bone marrow of breast cancer patients. Cancer Res..

[20] Yefenof E. (2008). Innate and Adaptive Immunity in the Tumor Microenvironment.

[21] Haanen J.B., Robert C. (2015). Immune checkpoint inhibitors. Prog. Tumor Res..

[22] Pico de Coana Y., Choudhury A., Kiessling R. (2015). Checkpoint blockade for cancer therapy. Revitalizing a suppressed immune system. Trends Mol. Med..

[23] Schirrmacher V. (2020). Cancer vaccines and oncolytic viruses exert profoundly lower side effects in cancer patients than other systemic therapies: A comparative analysis. Biomedicines.

[24] Schirrmacher V. (2015). Oncolytic Newcastle disease virus as a prospective anti-cancer therapy. A biological agent with potential to break therapy resistance. Expert Opin. Biol. Ther..

[25] Schirrmacher V., Van Gool S., Stuecker W. (2019). Breaking therapy resistance: An update on oncolytic Newcastle disease virus for improvements of cancer therapy. Biomedicines.

[26] Schirrmacher V., Stücker W., Lulei M., Bihari A.S., Sprenger T. (2015). Long-term survival of a breast cancer patient with extensive liver metastases upon immune and virotherapy: A case report. Immunotherapy.

[27] Schirrmacher V., Bihari A.S., Stücker W., Sprenger T. (2014). Long-term remission of prostate cancer with extensive bone metastases upon immuno- and virotherapy: A case report. Oncol. Lett..

[28] Van Gool S.W., Makalowski J., Bonner E.R., Feyen O., Domogalla M.P., Prix L., Schirrmacher V., Nazarian J., Stuecker W. (2020). Addition of multimodal immunotherapy to combination treatment strategies for children with DIPG: A single institution experience. Medicines.

[29] Schirrmacher V. (2016). Fifty years of clinical application of Newcastle disease virus: Time to celebrate!. Biomedicines.

[30] Schirrmacher V. (2020). New insights into mechanisms of long-term protective anti-tumor immunity induced by cancer vaccines modified by virus infection. Biomedicines.

[31] Deaton A., Cartwright N. (2018). Understanding and misunderstanding randomized controlled trials. Soc. Sci. Med..

[32] Vivot A., Jacot J., Zeitoun J.D., Ravaud P., Crequit P., Porcher R. (2017). Clinical benefit, price and approval characteristics of FDA-approved new drugs for treating advanced solid cancer, 2000–2015. Ann. Oncol..

[33] Mailankody S., Prasad V. (2014). Comparative effectiveness questions in oncology. N. Engl. J. Med..

[34] Booth C.M., Cescon D.W., Wang L., Tannock I.F., Krzyzanowska M.K. (2008). Evolution of the randomized controlled trial in oncology over three decades. J. Clin. Oncol..

[35] Tannock I.F., Amir E., Booth C.M., Niraula S., Ocana A., Seruga B., Templeton A., Vera-Badillo F. (2016). Relevance of randomized controlled trials in oncology. Lancet Oncol..

[36] Ioannidis J.P.A., Stuart M.E., Brownlee S., Strite S.A. (2017). How to survive the medical misinformation mess. Eur. J. Clin. Investig..

[37] Del Paggio J.C., Tannock I.F. (2019). The fragility of phase 3 trials supporting FDA-approved anticancer medicines: A retrospective analysis. Lancet Oncol..

[38] Davis C., Naci H., Gurpinar E., Poplavska E., Pinto A., Aggarwal A. (2017). Availability of evidence of benefits on overall survival and quality of life of cancer drugs approved by European Medicines Agency: Retrospective cohort study of drug approvals 2009-13. BMJ.

[39] Niraula S., Seruga B., Ocana A., Shao T., Goldstein R., Amir I.F.T. (2012). The price we pay for progress: A meta-analysis of harms of newly approved anticancer drugs. J. Clin. Oncol..

[40] Niraula S., Amir E., Vera-Badillo F., Seruga B., Ocana A., Tannock I.F. (2014). Risk of incremental toxicities and associeted costs of new anticancer drugs: A meta-analysis. J. Clin. Oncol..

[41] Johnson N., Bryant A., Miles T., Hogberg T., Cornes P. (2011). Adjuvant chemotherapy for endometrial cancer after hysterectomy. Cochrane Database Syst. Rev..

[42] Naci H., Davis C., Savović J., Higgins J.P.T., Sterne J.A.C., Gyawali B., Romo-Sandoval X., Handley N., Booth C.M. (2019). Design characteristics, risk of bias, and reporting of randomised controlled trials supporting approvals of cancer drugs by European Medicines Agency, 2014-16: Cross-sectional analysis. BMJ..

[43] Liu X., Tang L.L., Mao Y.P., Liu Q., Sun Y., Chen L., Lin J.-C., Ma J. (2019). Evidence underlying recommendations and payments from industry to authors of the National Comprehensive Cancer Network Guidelines. Oncologist.

[44] The Lancet Oncology (2019). Are results from clinical trials reliable?. Lancet Oncol..

[45] Tibau A., Molto C., Borrell M., Del Paggio J.C., Barnadas A., Booth C.M., Amir E. (2018). Magnitude of clinical benefit of cancer drugs approved by the US Food and Drug Administration based on single-arm trials. JAMA Oncol..

[46] Booth C.M., Del Paggio J.C. (2017). Approvals in 2016: Questioning the clinical benefit of anticancer therapies. Nat. Rev. Clin. Oncol..

[47] Jette A.M. (2006). Towards a Common Language for Functioning, Disability and Health. ICF: The International Classification of Functioning, Disability and Health. Phys. Ther..

[48] Collette L., Bogaerts J., Suciu S. (2012). Statistical methodology for personalized medicine. New developments at EORTC headquarters since the turn of the 21st century. Eur. J. Cancer Suppl..

[49] Rodon J., Soria J.C., Berger R., Batist G., Tsimberidou A., Bresson C., Lee J.J., Rubin E., Onn A., Schilsky R.L. (2015). Challenges in initiating and conducting personalized cancer therapy trials: Perspectives from WINTHER, a Worldwide Innovative Network (WIN) Consortium trial. Ann. Oncol..

[50] Catani J.P.P., Riechelmann R.P., Adjemian S., Strauss B.E. (2017). Near future of tumour immunology: Anticipating resistance mechanisms to immunotherapies, a big challenge for clinical trials. Hum. Vaccin. Immunother..

[51] Park J.J.H., Siden E., Zoratti M.J., Dron L., Harari O., Singer J., Lester R.T., Thorlund K., Mills E.J. (2019). Systematic review of basket trials, umbrella trials, and platform trials: A landscape analysis of master protocols. Trials.

[52] Schork N.J. (2015). Personalized medicine: Time for one-person trials. Nature.

[53] (2014). Design and Implementation of N-of-1 Trials: A User’s Guide.

[54] Ruhland S., Bartik B., Simiantonaki N., Schumacher J., Häcker B., Schumacher M., Schirrmacher V. (1997). Tumor-cell number and viability as quality and efficacy parameters of autologous virus-modified cancer vaccines in patients with breast or ovarian cancer. J. Clin. Oncol..

[55] Ockert D., Schirrmacher V., Beck N., Stoelben E., Ahlert T., Flechtenmacher J., Hagmüller E., Buchcik R., Nagel M., Saeger H.D. (1996). Newcastle disease virus-infected intact autologous tumor cell vaccine for adjuvant active specific immunotherapy of resected colorectal carcinoma. Clin. Cancer Res..

[56] Steiner H.H., Bonsanto M.M., Beckhove P., Brysch M., Geletneky K., Schuele-Freyer R.A., Kremer P., Ranaie G., Matejic D., Bauer H. (2004). Antitumor vaccination of patients with glioblastoma multiforme: A pilot study to assess feasibility, safety, and clinical benefit. J. Clin. Oncol..

[57] Karcher J., Dyckhoff G., Beckhove P., Reisser C., Brysch M., Ziouta Y., Helmke B.H., Weidauer H., Schirrmacher V., Herold-Mende C. (2004). Antitumor vaccination in patients with head and neck squamous cell carcinomas with autologous virus-modified tumor cells. Cancer Res..

[58] Schirrmacher V., Fournier P., Schlag P. (2014). Autologous tumor cell vaccines for post-operative active-specific immunotherapy of colorectal carcinoma: Long-term patient survival and mechanism of function. Expert Rev. Vaccines.

[59] Ellis R., Wollmann G., Schneider E.M., Aigner K., Brauns L., Nesselhut T., Ackva I., Weisslein C., Thaller A. (2020). Effective treatment of glioblastoma multiforme with oncolytic virotherapy: A case-series. Front. Oncol..

[60] Viciano A., Catanzano M. (2020). Günstig und Selbstgemacht. Report of the Süddeutsche Zeitung.

[61] Lieb K., Klemperer D., Ludwig W.D. (2011). Interessenkonflikte in Der Medizin.

[62] Lexchin J. (2018). Pharmaceutical company spending on research and development and promotion in Canada, 2013-2016: A cohort analysis. J. Pharm. Policy Pract..

[63] Sprenger T., Schirrmacher V., Stücker W., van Gool S.W. (2020). Position paper: New insights into the immunobiology and dynamics of tumor-host interactions require adaptations of clinical studies. Expert Rev. Anticancer Ther..

